# Development of Protein Rich Pregelatinized Whole Grain Cereal Bar Enriched With Nontraditional Ingredient: Nutritional, Phytochemical, Textural, and Sensory Characterization

**DOI:** 10.3389/fnut.2022.870819

**Published:** 2022-04-08

**Authors:** Farhana Mehraj Allai, B. N. Dar, Khalid Gul, Mohd Adnan, Syed Amir Ashraf, Md Imtaiyaz Hassan, Visweswara Rao Pasupuleti, Z. R. A. A. Azad

**Affiliations:** ^1^Department of Post-harvest Engineering and Technology, Faculty of Agricultural Science, Aligarh Muslim University, Aligarh, India; ^2^Department of Food Technology, Islamic University of Science and Technology, Awantipora, India; ^3^Department of Food Process Engineering, National Institute of Technology, Rourkela, India; ^4^Department of Biology, College of Science, University of Hail, Hail, Saudi Arabia; ^5^Department of Clinical Nutrition, College of Applied Medical Science, University of Hail, Hail, Saudi Arabia; ^6^Centre for Interdisciplinary Research in Basic Sciences, Jamia Millia Islamia, New Delhi, India; ^7^Department of Biomedical Sciences and Therapeutics, Faculty of Medicine and Health Sciences, University Malaysia Sabah, Kota Kinabalu, Malaysia; ^8^Department of Biochemistry, Faculty of Medicine and Health Sciences, Abdurrab University, Pekanbaru, Indonesia; ^9^Centre for International Collaboration and Research, Reva University, Rukmini Knowledge Park, Bangalore, India

**Keywords:** pregelatinized, antioxidants, textural properties, nutraceuticals, sensory attributes, dates

## Abstract

This study was aimed to use extrusion cooking as a pretreatment for non-conventional seeds (Indian horse chestnut flour) to blend them with whole grain flours (whole wheat flour, whole barley flour, and whole corn flour) for the development of a pregelatinized cereal bar (PCB). In this study, date paste (7.5–17.5%) and walnut grits (2.5–12.5%) were incorporated at varying levels to prepare PCB. The PCB was evaluated for its nutritional, color, textural (both three-point bending test and TPA), antioxidant activity, and sensory attributes. The flexural modulus, rupture stress, and fracture strain of PCB increased with the incorporation of a higher proportion of date paste. The protein and fiber content in PCB increased from 7.74 to 9.13% and 4.81 to 5.59% with the incorporation of walnut grits and date paste, respectively. The DPPH, total phenolic content, and water activity of PCB were determined, which progressively enhanced with increased levels of walnut grits and date paste. The correlation between sensory attributes and instrumental texture on PCB was also investigated. The correlation results showed a significant (*p* < 0.05) positive correlation between texture analysis and sensory hardness, springiness, adhesiveness, and negatively correlated to instrumental and sensory cohesiveness. For sensorial attributes, all PCB samples presented average scores of 7/10 and 4/5 for buying intention. Therefore, whole grain extrudates, date paste, and walnut grits can be efficiently used to develop PCB with improved nutritional, nutraceutical, and economic values.

## Introduction

The demand for ready-to-eat products has increased tremendously due to the change in consumers' lifestyles. In this context, a nutritious, healthy, balanced, and safe diet is always endorsed to reduce disorders such as diabetes, obesity, cardiovascular diseases, and malnutrition (1). Cereals play a vital role in the development of ready-to-eat snacks such as instant bars, energy bars, and cereal bars. The consumption of cereal products has been elaborated from the breakfast table till dinner in the form of flakes, rice, chapatti, or cereal bar as a snack (2). The increased intake of refined cereal-based products is due to their wide availability and low cost. However, during the milling process, bran and germ fractions are being removed, which results in the loss of many essential phytochemicals and macro as well as micronutrients in the human diet that have a direct relation with human health (3, 4). For this reason, consumers have valued the consumption of whole grains and their products as they contain all three fractions in the same proportion as present in the intact original grain (5). Cereal-based bars have become a significant part of the human diet, especially among children and can influence overall nutrition (6). They are convenient to carry, light in weight, available in small pouches, and can be consumed easily. Cereal bars are made primarily with whole grains such as whole wheat flour (WWF), whole corn flour (WCF), and whole barley flour (WBF). WWF contains high levels of dietary fiber and phenolic compounds including benzoxazinoids, lutein, zeaxanthin, and β-cryptoxanthin (7). WCF possesses a high concentration of zein and a prolamin protein fraction (8) and contains several bioactive constituents such as ferulic acids, anthocyanins, flavonoids, carotenoids, and phenolic compounds that have many different disease-preventing properties and potential health-promoting benefits (9). WBF is the richest source of tocols and is high in viscous soluble fiber especially β-glucan that lowers blood glucose serum, blood pressure, and low-density lipoprotein cholesterol (10) and also increases the intraluminal viscosity, thus extending gastric emptying time and absorption of nutrients in the small intestine (11). However, whole grain flours tend to have low mineral content and many other nutrients. As a result of this, the addition of Indian horse chestnut seed (non-conventional seeds), date, and walnut enhances the nutritional profile and the therapeutic value of developed whole grain-based cereal bars. Indian horse chestnut (*Aesculus indica*) has high levels of dietary fiber, starch, minerals, vitamins, and bioactive compounds (12). However, seeds are bitter in taste and poisonous, if consumed raw or without processing, due to anti-nutritional factors such as aesculin (saponins) and tannin. These anti-nutritional factors can be eliminated by carefully washing the seeds under running water (13). Dates are prolific with dietary fiber, protein, and carbohydrates mainly in the form of natural sugar (glucose, fructose, and sucrose) and are a rich source of minerals, vitamins, and bioactive compounds (14). It has potential medicinal values such as control or prevention of diabetes mellitus due to the presence of minerals and antioxidants. Children and women are more susceptible to the deficiency of nutritious food due to growth and reproduction, respectively (15). Phenolic compounds found in date paste can retard the α-amylase and α-glycosidase activities that also reduce the digestion rate of carbohydrates, resulting in less absorption of glucose into the blood circulation (16). Moreover, the texture of date paste is sticky and dense, which has a property of binding with other ingredients used during the product development. Walnut kernel stands out for high minerals, protein, vitamins, fat, and polyphenols. They also contain essential dietary fatty acids such as omega-3 and 6 polyunsaturated fatty acids that lower the risk of various disorders such as cholesterol, cardiovascular disease, and inflammation (17).

To develop nutritious healthy cereal bars, some preliminary trials were carried out. Among them, extrusion processing is recommended because of its versatility, which combines several different unit operations such as conveying, mixing, cooking, shearing, shaping, and forming in a single system that converts native ingredients present in cereals into a new functional product with unique shape and size (18). This technique manages to alter the molecular configuration of starch, leading to an increment in its functional characteristics (19). Recent literature has reported that extrusion cooking favors the synergic effect with starch that increases the viscoelasticity property of dough and also promotes the structural changes in ingredients such as in corn protein (zein) (20). In addition, there is ample research reporting other beneficial effects of extrusion cooking such as reduction or elimination of anti-nutritional factors, conversion of insoluble to soluble fiber, escalation of bioavailability of minerals and proteins, and increase in bioactive activity by releasing the phenolic compounds bound in insoluble fibers (21–23). In contrast, one drawback of pregelatinized cereal bar (PCB) developed from whole grain flours and Indian horse chestnut flour (IHCF) is texture and taste, but this can be reduced with the addition of date paste and walnut grits. These two natural ingredients additionally contribute to sweet taste and gritty mouthfeel and eliminate the filthy taste and texture of PCB. Therefore, this study was proposed to develop a whole grain-based cereal bar (from pregelatinized extrudates) incorporated with date paste and walnut grits. The functional cereal bar was analyzed for the nutritional, antioxidant, sensory, color, and textural characteristics. In addition, the correlation between instrumental texture and sensory attributes was established.

## Materials and Methods

### Procurement of Raw Materials

Whole grain wheat (SW-2), whole white corn (DT-2), and whole grain barley (PL 807) were obtained from Sher-e-Kashmir University of Agricultural Sciences and Technology, Shalimar, Jammu and Kashmir (J&K), and Kargil, India, respectively. Milling was done to obtain WWF, WCF, and WBF. The whole grain flours were then packed and stored at −21°C until further use. Indian horse chestnut seeds (*Aesculus indica*) were collected manually in October from the local area of Shalimar, J&K. Dates and walnuts were procured from a local market in Srinagar, J&K, India.

### Preparation of Indian Horse Chestnut Flour

The selected seeds were manually peeled, and the kernels were cut into two halves and then sliced using a vegetable slicer. The slices were blanched for 15 min and then soaked in water for 56 h followed by continuous washing and changing the water after every 2 h till the lather or foam comes down to remove the anti-nutritional factors (24). The slices were dried in a single layer in a tray drier (MFG, SSI-103C, Sood Steel Industries, India) at 60°C followed by cooling at room temperature and then fed to a laboratory grinder to obtain flour, which was passed through 60 mesh sieves.

### Preparation of Pregelatinized Cereal Bar

**Figure 1:**
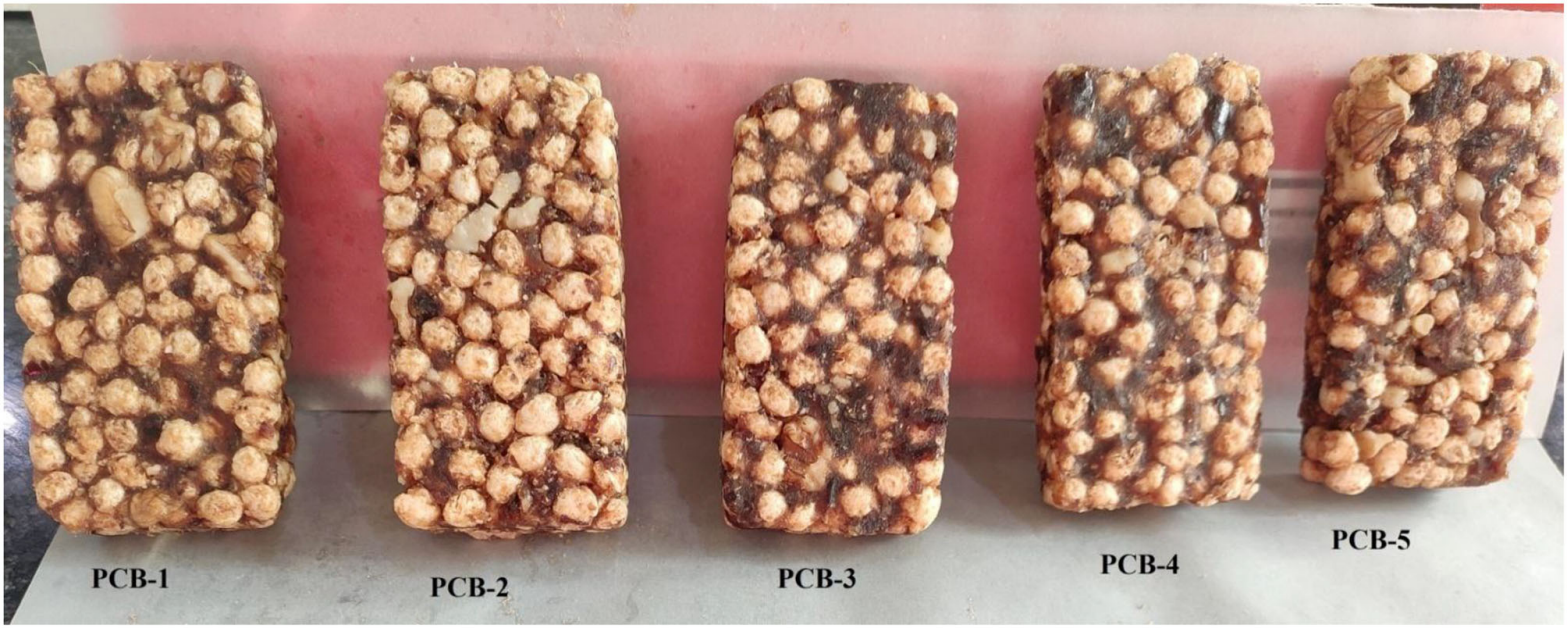
Images of pregelatinized whole grain-based cereal bar enriched with Indian horse chestnut flour, date paste, and walnut grits.

Cereal bars were prepared from extrudates made of WWF, WBF, WCF, and IHCF. Date paste and walnut kernels as walnut grits were also used for the development of whole grain PCBs. Extrudates were extruded through a corotating twin-screw extruder (Basic Technology Pvt. Ltd., Kolkata, India) with a die diameter of 3.0 mm and length to diameter ratio of 8:1. The process conditions of extruder and proportion of feed composition in the composite flour were designed by using Central Composite Rotational Design (CCRD). Feed moisture content (12–16%), barrel temperature (90–130°C), screw speed (320–380 rpm), and IHCF (2.5–4%) were the independent variables. The proportion of WWF and WBF was kept constant (10%, from preliminary trials) in all treatments. The remainder of 80% of the feed formulation was made of WCF in the control sample. IHCF (2.5–4%) was used to substitute WCF in the feed mixture. Numerical optimization was done to optimize the independent variables [IHCF (2.5%), moisture content (16%), barrel temperature (130°C), and screw speed (380 rpm)] to obtain the highest desirability and good quality extrudates. The novelty of this study was to use extrudates with their intact shape and size instead of using the flours as presented in Figure 1. Pitted dates were ground in a laboratory grinder (Usha-3345, New Delhi) to obtain date paste. Walnuts were deshelled and crushed to form walnut grits.

Extrudates were mixed with different percentages of date paste and walnut grits to obtain five different PCBs (PCB-1, PCB-2, PCB-3, PCB-4, and PCB-5) as shown in Table 1. The ingredients were individually weighed and mixed properly to get a uniform mixture. Molds of uniform size (8 × 4 × 1.5 cm) were used to develop a uniform PCB of different treatments (Table 1). Butter paper was used before filling up the mold. The bars were then dried in an oven at 40°C for 3 h and cooled to room temperature. PCBs were packed in aluminum foil polyethylene laminate and stored at 25°C for further analysis.

**Table 1:** Raw material and proportion used in the development of pregelatinized cereal bars.

**Ingredients**	**Formulations**				
	**PCB-1**	**PCB-2**	**PCB-3**	**PCB-4**	**PCB-5**
Extrudates (%)	80	80	80	80	80
Date Paste (%)	7.5	10	11.5	12.5	17.5
Walnut Grits (%)	12.5	10	8.5	7.5	2.5

*PCB, pregelatinized cereal bars; extrudates, made of 10% WWF, 10% WBF, 2.5% IHCF, and 77.5% WCF*.

### Nutritional Properties of Raw Material and Developed Pregelatinized Cereal Bars

The nutritional value of cereal bars, i.e., moisture, ash, fat, protein, and fiber, was evaluated according to the AOAC (25) and Sapna et al. (26) procedure. Carbohydrate content was determined by the difference method.


$$\begin{array}{l} Carbohydrate\;\left(\%\right)=\;100-(\%\;moisture+\%\;fat+\%\;protein\\ \;+\%\;ash+\%\;fiber) \end{array}$$

The calorific value was calculated using the equation:


$$\begin{array}{l} Energy\;value\left(\frac{Kcal}{100\;g}\right)=\left(4\times \%\;CHO\right)+\;\left(9\times \%\;fat\right)\\ \;+\;\left(4\times \%\;protein\right) \end{array}$$

### Water Activity (a_w_)

The water activity (a_w_) of cereal bars was evaluated using a water activity meter (Novasina AG CH-8853, Lachen) at 25°C. The analysis was done in triplicates.

### Color Evaluation

The color differences of cereal bars were analyzed by measuring the CIELAB space parameters using a Hunter Lab colorimeter (CR 300, Konica Minolta, Japan). Cereal bars were set in optical glass cells to measure the reflected color, represented as L^*^ (lightness/darkness), a^*^ (redness/greenness), and b^*^ (blueness/yellowness) values. Each value is an average of five different independent measurements.

### Antioxidant Activity

#### DPPH Radical Scavenging Activity

DPPH radical scavenging activity was estimated by a method discussed in the study by Kaur et al. (27). Extraction of samples was done in 80% methanol at 25°C for 120 min. After extraction, the samples were filtered through Whatman No. 1 filter paper. From the extracts, an aliquot of 100 μl was taken and added to 3.9 ml of DPPH. After 30 min of incubation, absorbance was measured at 517 nm at room temperature.


$$\begin{array}{l} DPPH\;radical\;scavanging\;activity\;\left(\%\right)=\;\frac{A_{C}-A_{e}}{A_{C}}\times 100 \end{array}$$

where A_c_ is the absorbance of control at 0 min time and A_e_ is the absorbance of the sample at 30 min.

### Total Phenolic Content

The procedure developed by Zahoor and Khan (28) was used to calculate the TPC in raw and PCB samples. Methanol as solvent was used for the extraction process. Samples of 2 g were homogenized in 20 ml of methanol. The homogenate was then kept undisturbed for 12 h. The obtained mixture was centrifuged at 10,000 × *g* for 15 min. After centrifugation, 0.2 ml of aliquot is mixed with 1.5 ml of Folin-Ciocalteu reagent and 1.2 ml of 7.5% of Na_2_CO_3_. The mixture was then kept aside for 2 h at 25°C. Finally, the absorbance was measured using a spectrophotometer at 765 nm. A calibration curve was made by gallic acid, and the TPC was expressed as mg gallic acid equivalents (GAE)/g of dry sample.

### Texture Analysis

Texture analysis was done using the following two methods.

#### Three-Point Bending Test

**Figure 2:**
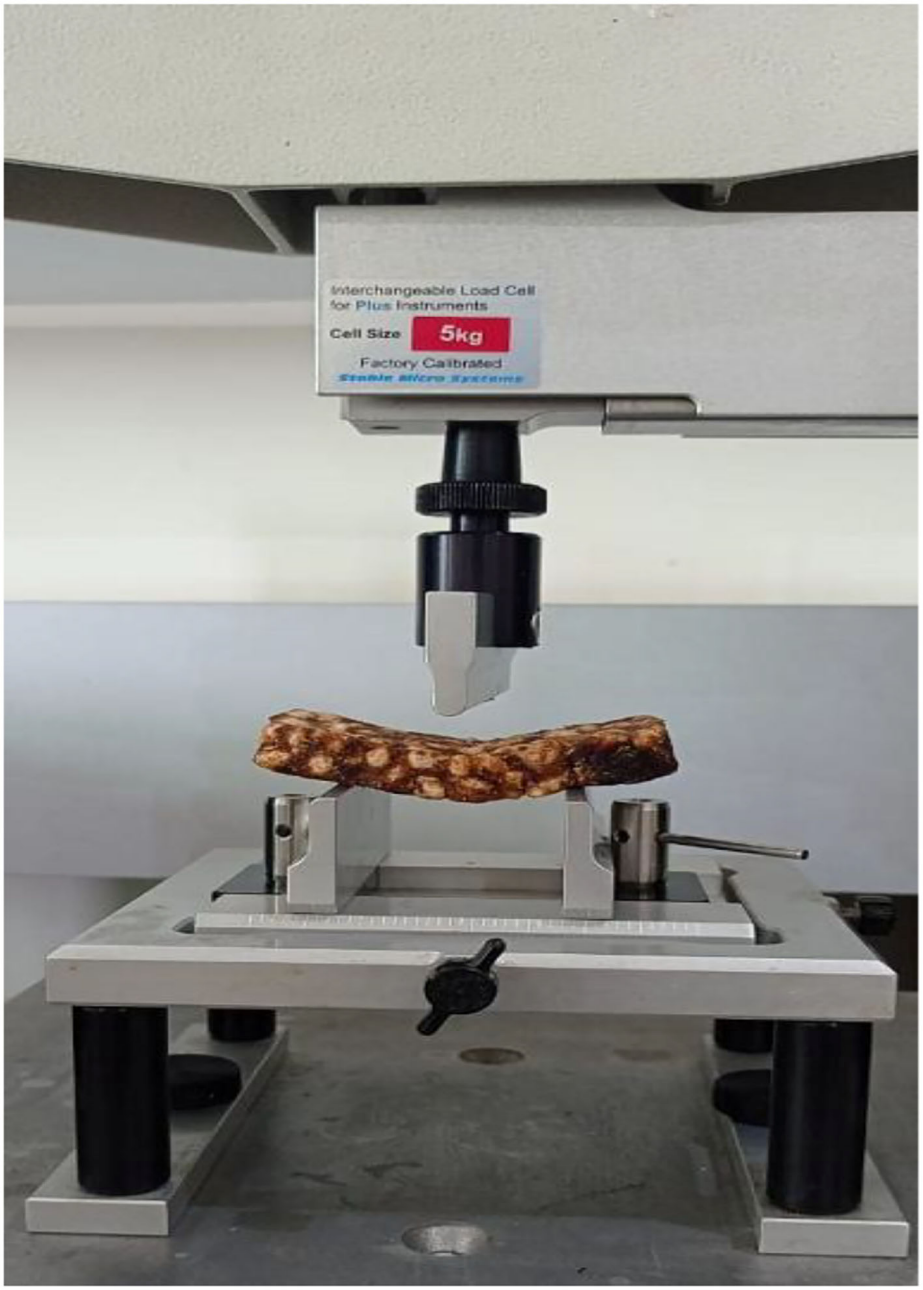
Three point bending rig test.

The texture of the cereal bars was evaluated using TA-HD Plus, texture analyzer (Stable Micro System, Godalming, Surrey, UK) equipped with a load cell of 50 N and a three-point bending rig (Figure 2). A PCB is placed between two supports of width 3.25 mm with a fixed distance between the two supports (L) that is predetermined based on the sample size. The sample is compressed vertically downwards at the center of the cereal bar, with a blunt blade probe (HDP/3PB) (40 mm) at a crosshead speed of 1 mm/s to obtain maximum compression force (F) (N, Newton) or force deformation curve and is termed as “Force of rupture” (29).

The flexural modulus (E), rupture stress (σ), and fracture strain (r) of the samples were derived using the following equation:


$$\begin{array}{l} \sigma =F\frac{3L}{2h^{2}w}\\ E=\;\frac{F}{d}\frac{L^{3}}{4h^{3}w}\\ \;r=\;\frac{6Dw}{L^{2}} \end{array}$$

where F (N) is the force of rupture, h (mm) is the thickness of the bar, w (mm) is the width of the bar, F/d (N/mm) is the slope of the linear part of a force-displacement curve, and D (mm) is the deflection of the center of the bar at the point of the break.

#### Texture Profile Analysis

Texture profile analysis of PCB samples was determined by Carvalho and Conti-Silva's (30) method with a slight modification. The TPA technique involves a two-cycle compression test, which imitates two bites. The instrument is equipped with a 30 mm cylindrical probe (P/36 R) and operated at a pretest speed of 1 mm/s, test speed of 0.50 mm/s, posttest speed of 10.00 mm/s, trigger force of 5.0 g, and time of 5 s were kept in between the two compressions. The samples were compressed to 50% of their original height. The result represented the hardness, springiness, gumminess, adhesiveness, and cohesiveness parameters.

### Sensory Evaluation

The sensory evaluation was performed in the Department of Food Science and Technology, Islamic University of Science and Technology, Srinagar, India, in individual cabins. A total of 35 judges, who were potential consumers of the product, were randomly selected from the campus. Samples were given in a coded form with four-digit numbers and a 9-point hedonic scale (1 = dislike very much and 9 = like very much) was used to carry out the acceptance test (31) for aroma, color, texture, taste, and overall acceptability. After every sample, the consumers were guided to rinse their mouth with water, to differentiate the taste of different treatments of cereal bars. Acceptance results were complemented by questioning the purchase intent regarding each sample, using the 5-point scale (5 = definitely would buy, 3 = might or might not buy, and 1 = definitely would not buy). The acceptability index (AI) was evaluated using the following equation:


$$\begin{array}{l} AI\;\left(\%\right)=\;\frac{A}{B}\times 100 \end{array}$$

where A and B denote the average score given to the product and the maximum score obtained for the product, respectively. AI with a 70% score is considered to be a good product (32).

### Statistical Analysis

The data collected from the experiments were subjected to SPSS (version 20) statistical software package. Values were expressed as mean ± SD. The significant difference at (*p* < 0.05) was performed by one-way analysis of variance (ANOVA) and Duncan's multiple range test.

## Results and Discussion

### Proximate Composition and Color Analysis of Raw Materials

The proximate composition of whole grain-based extrudates (WGE), date paste, and walnut grits are shown in Table 2. The moisture, protein, ash, fat, crude fiber, and carbohydrate content of WGE utilized in this study were 3.59, 9.01, 1.67, 4.5, 4.91, and 76.32%, respectively. The protein content of walnut grits (14.38%) and date paste (2.4%) plays a significant role in improving the nutritional quality of PCBs (33, 34). Walnut grits had higher fat content (10.03%) but lower fiber content (2.37%) than date paste (9.6%) and WGE (4.91%). The DPPH and TPC of raw materials, i.e., whole grain extrudates, date paste, and walnut grits ranged from 3.56 to 23.71% and 5.87–232.01 mg GAE/g, respectively. Therefore, the addition of date paste and walnut grits to WGE will increase the antioxidant activity, dietary fatty acids, amino acid profile, and dietary fiber of the developed product.

**Table 2:** Proximate and color analysis of extrudates, date paste, and walnut grits.

**Parameters analyzed**	**Extrudate**	**Date paste**	**Walnut grits**
Moisture (%)	3.59 ± 0.08^c^	18.3 ± 0.26^a^	3.91 ± 0.27^b^
Protein (%)	9.01 ± 0.12^b^	2.4 ± 0.12^c^	14.38 ± 0.28^a^
Fat (%)	4.5 ± 0.08^b^	0.8 ± 0.02^c^	10.03 ± 0.16^a^
Crude fiber (%)	4.91 ± 0.09^b^	9.6 ± 0.5^a^	2.37 ± 0.16^c^
Ash (%)	1.67 ± 0.07^a^	1.61 ± 0.05^a^	1.66 ± 0.09^a^
Carbohydrate (%)	76.32 ± 0.21^a^	67.3 ± 0.33^b^	67.65 ± 0.14^c^
DPPH (%)	18.33 ± 0.17^b^	3.56 ± 0.21^c^	23.71 ± 0.17^a^
TPC mg GAE/g	5.87 ± 0.22^c^	232.01 ± 0.15^a^	44.53 ± 0.09^b^
L*	64.9 ± 0.15^a^	43.31 ± 0.13^c^	58.5 ± 0.07^b^
a*	3.68 ± 0.02^b^	12.03 ± 0.17^a^	1.78 ± 0.13^c^
b*	14.97 ± 0.33^b^	5.62 ± 0.24^c^	35.2 ± 0.22^a^

Extrudates, made of 10% WWF, 10%WBF, 2.5% IHCF, and 77.5% WCF; TPC, total phenolic content; DPPH, radical scavenging activity; L*, lightness; a*, redness; b*, yellowness.

*Values are an average of triplicate observations (± SD). Values followed by similar superscripts in rows do not differ significantly (p ≤ 0.05)*.

The color characteristics of extrudates, date paste, and walnut grits are depicted in Table 2. The L^*^ value indicates the lightness was higher for WGE (64.9) and walnut grits (58.5) as compared with date paste (43.31). Date paste showed more redness (a^*^ = 12.03) than WGE (3.68) and walnut grits (1.78). The yellowish color was more prominent in walnut grits (35.2) indicated by its b^*^ values followed by WGE (14.97) and date paste (5.62).

### Nutritional Composition of Pregelatinized Cereal Bar

Results for the nutritional composition of PCBs are provided in Table 3. The study reported that with the substitution of date paste and walnut grits, the nutritional profile of formulated PCB improved. Moisture content in PCB is an important parameter as it not only affects the shelf life but also affects the quality of a product such as taste, texture, and appearance. Water content values of PCB were significantly (*p* ≤ 0.05) lower than date paste (18.3%) but higher than walnut grits (3.9 %). This result follows the previous observations of Nadeem et al. (35) and Yerlikaya et al. (36) who have worked on date bar and walnut composition, respectively. It was found that moisture content ranged between 8.7 and 11.25%. The highest moisture content was found in PCB-5 (11.25%), while the lowest value was found in PCB-1 (8.7%). The increase in moisture content reflects the increased residual water content of the product's development from the protein enrichment ingredient (37, 38). PCB-2 showed significantly higher protein (9.13%), fat (4.71%), and ash content (1.76%) with an increase in walnut grits. Walnut proteins contain a relatively higher quantity of arginine and also contain a myriad of essential amino acids such as albumin, glutelin, and globulin (39). Thus, walnut proteins could be a good source of essential amino acids for both kids and adults. In date paste, the amount of protein content is too low to be considered, but still, it can fulfill the daily requirements of the human body (40). Generally, walnut kernels contain 62–68% of oil mainly composed of linoleic, oleic, and linolenic acids. These fatty acids have different health-promoting benefits and are vital to the nutritional and economic value of food products (41). Walnut grits are perfect ingredients for products that do not need further cooking such as bars, muffins, and cakes as high content of linoleic acid on cooking are more prone to charring (42).

**Table 3:** Nutritional values of developed PCB.

**Sample**	**Moisture (%)**	**Protein (%)**	**Fat (%)**	**Crude fiber (%)**	**Ash (%)**	**Carbohydrate (%)**	**Energy value (Kcal/100 g)**
PCB-1	8.7 ± 0.25^e^	9.04 ± 0.05^b^	4.64 ± 0.03^b^	4.81 ± 0.07^e^	1.72 ± 0.03^b^	71.09 ± 0.09^a^	362.28 ± 0.21^a^
PCB-2	9.1 ± 0.03^d^	9.13 ± 0.12^a^	4.71 ± 0.06^a^	5.30 ± 0.09^c^	1.76 ± 0.02^a^	70.06 ± 0.27^b^	359.15 ± 0.33^b^
PCB-3	10.2 ± 0.29^c^	8.36 ± 0.08^d^	4.31 ± 0.02^d^	5.12 ± 0.07^d^	1.67 ± 0.04^c^	70.34 ± 0.22^b^	353.59 ± 0.16^c^
PCB-4	10.62 ± 0.08^b^	8.59 ± 0.05^c^	4.58 ± 0.08^c^	5.40 ± 0.08^b^	1.70 ± 0.02^b^	69.11 ± 0.31^c^	352.02 ± 0.22^d^
PCB-5	11.25 ± 0.14^a^	7.74 ± 0.11^e^	3.95 ± 0.07^e^	5.59 ± 0.08^a^	1.68 ± 0.04^c^	69.79 ± 0.29^c^	345.67 ± 0.11^e^

*PCB, pregelatinized cereal bar*.

*Values are an average of triplicate observations (± SD). Values followed by similar superscripts in columns do not differ significantly (p ≤ 0.05)*.

Concerning crude fiber, PCB-5 prepared from a higher percentage of date paste showed significantly higher content (5.59%) compared with PCB-4, PCB-3, PCB-2, and PCB-1. A prominent increase in crude fiber may be ascribed to the extrudates made of whole grains and date paste as it contains a higher number of polysaccharides composed of galactan, glucan, arabian, xylan, cellulose, hemicelluloses, pectin, etc. (43).

The carbohydrate content of PCB varied from 69.11 to 71.09% (Table 2). In this study, the results were comparable with the high carbohydrate content generally present in bars developed from cereal and fruits, cereal bars with fruit by-products such as guava peels and cashew, cereal bars with tonka beans, and gluten-free cereal bars. A previous study has shown that cereal bars prepared from puffed rice, fruits, and cereals contain increased content of carbohydrates (44). Additionally, cereal bars made with the substitution of sugar and honey as a binder reported high content of carbohydrates (45). The gross energy values of PCB ranged between 345.67 and 362.28 kcal/100 g (Table 2). The increase in energy values of developed PCB can be possibly due to the subsequent increment in its carbohydrate content. These results coincide well with the results reported by Samakradhamrongthai et al. (31).

### Antioxidant Activity and TPC

The DPPH radical scavenging activity and TPC of PCB are presented in Table 4. PCB -1 had the highest DPPH content among all the tested samples with a DPPH of 15.48%. Similarly, PCB-5 showed the highest amount of TPC (127.23 mg GAE/g). After extrusion, the DPPH and TPC of samples were reduced since the phenolic compounds are heat sensitive. In addition, the higher shear force might cause a breakdown of the molecular structure of bioactive compounds, resulting in the decrease of phenolic content. This result was consistent with the findings of Bhat et al. (46) and Cheng et al. (47). The addition of date paste and walnut grits to extrudates increased the DPPH and TPC up to 3 and 2.5 times, respectively. The higher antioxidant potential and TPC in PCB may be ascribed to the bioactive rich compounds in date paste such as phytosterols, anthocyanin, phenolics, tocopherols, carotenoids, tocotrienols, and dietary fiber (48) and walnut grits such as ellagitannins, melatonin, and serotonin (49). Similar results of elevated antioxidant properties were reported by Kaur et al. (27) for pasta products incorporated with orange peel powder and cucumber peel powder.

**Table 4:** Water activity, color analysis, DPPH, and total phenolic content of pregelatinized cereal bar enriched with date paste and walnut kernels.

**Sample**	**a_**w**_**	**Color analysis**			**Antioxidant activity**	
		L*	a*	b*	**DPPH (%)**	**TPC (mg GAE/g)**
PCB-1	0.344 ± 0.001^e^	57.22 ± 2.5^a^	11.15 ± 0.05^a^	22.80 ± 0.07^b^	15.48 ± 0.19^a^	49.27 ± 0.23^e^
PCB-2	0.411 ± 0.009^d^	47.64 ± 0.4^b^	10.64 ± 0.11^b^	23.91 ± 0.11^a^	10.32 ± 0.17^c^	82.34 ± 0.33^c^
PCB-3	0.438 ± 0.016^c^	44.80 ± 2.6^c^	9.12 ± 0.11^c^	21.36 ± 0.11^c^	8.53 ± 0.27^d^	66.26 ± 0.024^d^
PCB-4	0.500 ± 0.004^b^	43.62 ± 1.6^d^	9.07 ± 0.08^d^	21.45 ± 0.06^d^	12.11 ± 0.15^b^	109.77 ± 0.17^b^
PCB-5	0.593 ± 0.006^a^	40.02 ± 0.1^e^	8.37± 0.08^e^	20.08 ± 0.08^e^	5.32 ± 0.21^e^	127.23 ± 0.11^a^

PCB, pregelatinized cereal bar; a_w_, water activity; L*, lightness; a*, redness; b*, yellowness.

*Values are an average of triplicate observations (± standard deviation). Values followed by similar superscripts in columns do not differ significantly (p ≤ 0.05)*.

### Water Activity

The water activity (a_w_) of PCB varied from 0.34 to 0.59 (Table 3). It measures the free water in foods. PCB-5 reported the highest a_w_ (0.593), which contained the highest percentage of date paste (17.5%). The lowest a_w_ (0.34) was observed in PCB-1, which had the lowest quantity of date paste (5%). The a_w_ below a critical value of 0.6 is recommended for safe storage (50). Generally, cereal bars developed with the addition of sugar observed a_w_ in a range of 0.1–0.6, whereas cereal bars formulated without the substitution of sugar reported a_w_ >0.7 (31). These results indicate that the food products can be stored for a long time without the risk of production of mycotoxins and microbial growth, which would lead to spoilage (51).

### Color Analysis

Color is an important parameter to decide the acceptability of a food product. The results obtained for color analysis were found to be ranged from 40.02 to 57.22, 8.37 to 11.15, and 20.08 to 23.89 for L^*^, a^*^, and b^*^ values, respectively. The color values (L^*^, a^*^, and b^*^) were mainly influenced by the walnut grits and date paste. All the color values showed statistically significant (*p* < 0.05) variations among different formulations (Table 3). The highest value (57.22) for L^*^ was observed in PCB-1, while the lowest value (40.02) was reported in PCB-5. L^*^ values differed significantly with an increased percentage of date paste that showed a slightly darker color (lower lightness values). a^*^ and b^*^ represent the red-green axis and blue-yellow axis, respectively. All the samples showed positive a^*^ and b^*^ values and therefore indicate the reddish and yellowish color of the developed product due to the presence of date paste and walnut grits (52).

### Textural Analysis

#### Three-Point Bending of Pregelatinized Whole Grain Cereal Bar

Three-point bending parameters such as flexural modulus (E), rupture stress (σ), and fracture strain (r) are shown in Table 5. Flexural modulus (E) also known as bending modulus indicates the stiffness of a product, i.e., the higher the flexural modulus of a product, the harder is to bend. Generally, samples with higher moisture content showed increased modulus, rupture stress, and fracture strain (29) as excess water acts as a plasticizer in amorphous regions of starch molecules, leading to the breakdown of hydrogen bonds and the formation of bonds between associated starch chain and water molecules (53). In addition, an increase in water content decreases the viscosity, resulting in a hard, compressed, and dense structure of the product (54). Sample PCB-5 showed higher bending modulus (15.86 MPa) due to the high percentage of date paste (17.5 %), i.e., as the date paste contains natural sugar that has a plasticizing effect even at a low percentage led to lower melt temperature and decreased water vapor pressure resulting in the hard texture of the product. Another explanation results from the difference in the properties of ingredients used during processing such as walnut tend to return to their original shape after compression (55). Thus, the ingredients exhibited different functional and mechanical characteristics depending on the process conditions.

**Table 5:** Textural characteristics of developed PCB.

**Samples**	**3-point bending test**			**Textural profile analysis**				
	**σ (MPa)**	**E (MPa)**	**r (MPa)**	**Hardness (N)**	**Springiness**	**Adhesiveness**	**Cohesiveness**	**Gumminess (N)**
PCB-1	4.33 ± 0.11^d^	8.05 ± 0.09^e^	0.67 ± 0.04^e^	82.64 ± 0.22^e^	0.16 ± 0.17^d^	−835.77 ± 0.07^a^	0.126 ± 0.34^d^	14.41 ± 0.22^e^
PCB-2	6.04 ± 0.3^c^	9.23 ± 0.25^d^	0.71 ± 0.27^d^	109.15 ± 0.19^d^	0.18 ± 0.08^c^	−1,107.3 ± 0.05^b^	0.136 ± 0.11^c^	18.84 ± 0.18^d^
PCB-3	6.07 ± 0.14^c^	10.73 ± 0.22^c^	0.94 ± 0.31^c^	122.34 ± 0.35^c^	0.21 ± 0.26^b^	−1,248.3 ± 0.17^c^	0.139 ± 0.16^c^	21.0 ± 0.09^c^
PCB-4	9.12 ± 0.09^b^	12.34 ± 0.17^b^	1.44 ± 0.24^b^	134.62 ± 0.32^b^	0.24 ± 0.09^b^	−1,777.9 ± 0.04^d^	0.147 ± 0.11^b^	29.78 ± 0.04^b^
PCB-5	10.95 ± 0.16^a^	15.86 ± 0.19^a^	1.78 ± 0.23^a^	147.74 ± 0.35^a^	0.42 ± 0.27^a^	−3,171.1 ± 0.11^e^	0.153 ± 0.17^a^	32.6 ± 0.21^a^

*PCB, pregelatinized cereal bars; flexural modulus (E); rupture stress (σ); fracture strain (r)*.

*Values are an average of triplicate observations (± SD). Values followed by similar superscripts in columns do not differ significantly (p ≤ 0.05)*.

Additionally, a high amount of date paste and walnut can increase the rupture stress (σ) and fracture strain (r) of whole grain PCB from 4.33 to 10.95 (MPa) and 0.67 to 1.78 (MPa), respectively, due to the increment in moisture absorption of sucrose and liquid sugar from date paste that shows elevated hygroscopic nature in a cereal bar. The incorporation of sweetener or binder can influence the solid cohesion between the ingredients used during the development of cereal bar due to strong interaction between sugar networks that needs high penetration forces. Similar results were found in the development of granular and cereal bars by using sucrose as an alternative sweetener (56).

#### Texture Profile Analysis

Pregelatinized whole grain cereal bar contains different percentages of protein-enriched ingredients date paste and walnut (Table 5). Results obtained by TPA showed significant variation (*p* < 0.05) for the formulation of PCB-1, PCB-2, PCB-3, PCB-4, and PCB-5. Variation in different parameters of TPA could be ascribed to different moisture content and concentration of ingredients.

Hardness values increased with an increasing percentage of date paste. Highest value for hardness was found in PCB-5 (147.74 N) followed by PCB-4 (134.62 N), PCB-3 (122.34 N), PCB-2 (109.15 N), and PCB-1 (82.64 N). These values were measured from the maximum force obtained during the first probe penetration. The higher amount of date paste can increase the hardness due to the moisture migration between carbohydrates such as starches, sugars, dietary fibers, and proteins that make food products less elastic and more prone to rupture upon compression (57), i.e., the transformation of rubbery behavior (easy to deform) of date paste to leathery state (tough to deform) as the water molecules in date paste migrates from protein toward sugars, which requires higher penetration force. When all the ingredients such as extrudates (made of a mixture of WWF, WBG, WCF, and IHCF), walnuts, and date paste are added together, it caused the stickiness and hardness to increase as shown in Table 5. Thus, the result suggested that the amount of protein and type of fiber also affects the hardness of the cereal bars (58).

Cohesiveness and adhesiveness are related to the probe withdrawal force within the PCB. Cohesiveness values were 0.126, 0.136, 0.139, 0.147, and 0.153 for PCB-1, PCB-2, PCB-3, PCB-4, and PCB-5, respectively. Cohesiveness indicates the strength of intrinsic interactions that shows the degree of mass of ingredients sticks together after chewing (59). Thus, when an increased percentage of date paste is mixed with other ingredients, more cohesion was observed. This result suggests that increased heterogeneity between raw materials leads to more interactions and thus higher cohesiveness strength. Similar results were reported by Conte et al. (60) for gluten-free bread. Adhesiveness determines the ratio of work to force that overcomes the attractive force between the surface of a probe and the product (61). Negative values of adhesiveness (Table 5) show that the bars made from date paste are very sticky or adhesive (33). Bars from the PCB-5 recipe were more cohesive and adhesive, but the sample PCB-1 and PCB-2 had a gritty mouthfeel as it contains more walnut grits than other bars.

Springiness values of PCB varied from 0.16 to 0.42, this means that the values for springiness are <1, suggesting that the bars developed from date paste do not come back to their original shape once the force is applied to them (33). Gumminess values were calculated between cohesiveness with hardness and are defined by the force needed to break the sample completely into a steady state of swallowing (62). Gumminess values were 14.41 N, 18.84 N, 21, 29.78 N, and 32.6 N for PCB-1, PCB-2, PCB-3, PCB-4, and PCB-5, respectively. Since PCB-5 and PCB-4 have the higher values due to the inclusion of a higher percentage of date paste and walnut that increased the number of chews required before swallowing.

### Sensory Evaluation

The mean values for sensorial evaluation are depicted in Table 6. All the samples showed good scores for taste, texture, color, and aroma of all the parameters analyzed. The average values ranged between 7.54 and 8.0, which means “like slightly” to “like very much” in terms of hedonic scale. The test for an index of purchase indicates the probable buying of a product. PCB-2, PCB-3, PCB-4, and PCB-5 showed non-significant variations, and the panelist suggested that the products are recommended to buy (4 scores). The acceptability index (AI) was determined based on the mean scores given by the judges, where PCB-5 (containing 12.5% of date paste and 7.5% of walnut grits) reported the highest AI (79.45%), but other treatments showed AI above 70% (Table 6). A product having at least 70% of approval is considered to be acceptable (63).

**Table 6:** Sensory and average acceptance of PCB.

**Samples**	**Taste**	**Texture**	**Color**	**Aroma**	**Overall acceptability**	**Intention to purchase**	**Acceptability index (%)**
PCB-1	7.55 ± 0.23^d^	7.10 ± 0.11^c^	8.00 ± 0.05^a^	7.00 ± 0.03^b^	7.54 ± 0.04^d^	3.00^b^	75.22^d^
PCB-2	7.92 ± 0.14^b^	7.05 ± 0.15^c^	7.56 ± 0.18^c^	7.10 ± 0.02^b^	7.88 ± 0.21^b^	4.00^a^	77.23^c^
PCB-3	7.77 ± 0.07^c^	7.05 ± 0.23^c^	7.94 ± 0.25^b^	7.10 ± 0.16^b^	7.63 ± 0.23^c^	4.00^a^	70.11^e^
PCB-4	7.8 ± 0.05^c^	7.81 ± 0.19^b^	7.00 ± 0.33^d^	8.00 ± 0.19^a^	7.9 ± 0.19^a^	4.00^a^	79.45^a^
PCB-5	8.00 ± 0.09^a^	8.00 ± 0.25^a^	6.90 ± 0.36^d^	8.04 ± 0.09^a^	8.00 ± 0.07^a^	4.00^a^	79.01^b^

*PCB, pregelatinized cereal bars*.

*Values are an average of triplicate observations (± SD). Values followed by similar superscripts in columns do not differ significantly (p ≤ 0.05)*.

### Correlation Between Human Assessment and Mechanical Variable

The Pearson correlation coefficient was used to analyze the correlation between two variables, i.e., human assessment (sensory analysis) and mechanical variable (instrumental texture). In a correlation coefficient, a perfect linear relationship indicates an absolute value of 1, and a *p*-value < 0.05 shows that the parameters were significantly related to each other. The behavior of date paste and walnut grits in PCB were achieved through developing the correlations of the parameters obtained by human assessment and mechanical variable as given in Table 7.

**Table 7:** Correlation coefficient between human assessment and mechanical variable.

**Mechanical variable**	**Human assessment**	**Pearson's coefficient correlation**	***p*-value**
Hardness	Firmness	0.945	0.004
Cohesiveness	Breakdown rate	−0.932	0.017
Adhesiveness	Adhesive (mouth)- degree to which PCB sticks to the teeth surface after swallowing	0.967	0.002
Springiness	Sample recovery –recoverable strain	0.8	0.24

From the analysis, hardness and springiness (mechanical) showed an excellent correlation with firmness and sample recovery (human assessment). There is a significant positive (*p* < 0.01) correlation between sensory and instrumental hardness as well as sensory and instrumental springiness. These results confirm the usefulness of TPA in predicting the human perception (sensory) of cereal bars, as reported in a previous study (64). A PCB sample with minimum hardness value obtained by mechanical variables could be translated to less firmness in the mouth, which needs minimum efforts to break down the sample. In the instrumental texture test, a sample is first compressed where the sample surface is less than the instrumental surface. In sensory, deformation of sample is done either by fingers or teeth, i.e., the force required to compress the food product between molar teeth during mastication (65). Springiness can be defined as the recovery deformation. It is the function of test time, less test time means an elastic property, and therefore, the textural values are close to 1. Mechanical springiness is a good evaluator of sensory recovery deformation (66).

Sensory adhesiveness (mouth) shows a positive correlation with mechanical adhesiveness (the rate at which the sample adheres to the probe surface after the first compression), while a negative relationship was observed between sensory cohesiveness (breakdown) and instrumental cohesiveness. Table 7 depicts that adhesiveness values from both human perception and mechanical were strongly correlated (*r* = 0.96) and poor correlation was between sensory and instrumental cohesiveness (*r* = −0.932) (67). For dry samples such as cereal bars, an adequate amount of saliva is mixed during chewing and before swallowing. Thus, it would be difficult to determine the accurate cohesiveness by instrumental test unless the effect of incorporation of saliva is added to it. Cohesiveness increases with the presence of saliva as it enhances the viscoelastic characteristic of food due to mucin components (68).

The results obtained for PCB incorporated with date paste and walnut grits showed that instrumental measurements are one of the best methods to evaluate most of the human assessment or sensory attributes. It is a cost-effective and rapid tool that mimics oral processing or sensory evaluation.

## Conclusion

Utilizing extrudates, date paste, and walnut grits to develop PCB is an excellent way to attract consumers. Incorporating date paste and walnut grits enhanced the three-point bending test and textural properties. The antioxidants, protein, ash, fiber, and dietary fatty acid content progressively increased with increased levels of date paste and walnut grits. High acceptability index and appealing color for all PCB were obtained. Instrumental measurements and sensory attributes were positively correlated. Hardness, adhesiveness, and springiness attributes from the instrument and sensory attributes were positively correlated. Cohesiveness obtained from TPA was negatively correlated to the breakdown rate. It indicates protein-rich PCB is easy to chew into a desirable state before swallowing, which makes it suitable for kids and elderly people. This novel PCB can be consumed as ready-to-eat food, a healthy snack bar, and as a dessert (if kept chilled). Developing a healthy product from date paste and walnut kernels rich in bioactive compounds, proteins, and dietary fiber is a practical approach that enhances the potential health-promoting benefits.

## Data Availability Statement

The original contributions presented in the study are included in the article/supplementary material, further inquiries can be directed to the corresponding authors.

## Author Contributions

FA: conceptualization, writing—original draft preparation, data curation, investigation, and methodology. BD: data analysis, validation, and visualization. KG: formal analysis, supervision, investigation, and validation. MA: data curation, validation, and writing—review and editing. SA: methodology and writing—review and editing. MH: conceptualization, writing—original draft preparation, and investigation. VP and ZA: conceptualization, data analysis, validation, project administration, and writing—review and editing. All authors contributed to the article and approved the submitted version.

## Conflict of Interest

The authors declare that the research was conducted in the absence of any commercial or financial relationships that could be construed as a potential conflict of interest.

## Publisher's Note

All claims expressed in this article are solely those of the authors and do not necessarily represent those of their affiliated organizations, or those of the publisher, the editors and the reviewers. Any product that may be evaluated in this article, or claim that may be made by its manufacturer, is not guaranteed or endorsed by the publisher.
